# Targeting BTK for the treatment of FLT3-ITD mutated acute myeloid leukemia

**DOI:** 10.1038/srep12949

**Published:** 2015-08-21

**Authors:** Genevra Pillinger, Amina Abdul-Aziz, Lyubov Zaitseva, Matthew Lawes, David J. MacEwan, Kristian M. Bowles, Stuart A. Rushworth

**Affiliations:** 1 Department of Molecular Haematology, Norwich Medical School, University of East Anglia, Norwich Research Park, Norwich, NR4 7TJ, United Kingdom; 2 Department of Molecular and Clinical Pharmacology, Institute of Translational Medicine, University of Liverpool, Liverpool, L69 3GE, United Kingdom; 3 Department of Haematology, Norfolk and Norwich University Hospitals NHS Trust, Colney Lane, Norwich, NR4 7UY, United Kingdom

## Abstract

Approximately 20% of patients with acute myeloid leukaemia (AML) have a mutation in FMS-like-tyrosine-kinase-3 (FLT3). FLT3 is a trans-membrane receptor with a tyrosine kinase domain which, when activated, initiates a cascade of phosphorylated proteins including the SRC family of kinases. Recently our group and others have shown that pharmacologic inhibition and genetic knockdown of Bruton’s tyrosine kinase (BTK) blocks AML blast proliferation, leukaemic cell adhesion to bone marrow stromal cells as well as migration of AML blasts. The anti-proliferative effects of BTK inhibition in human AML are mediated via inhibition of downstream NF-κB pro-survival signalling however the upstream drivers of BTK activation in human AML have yet to be fully characterised. Here we place the FLT3-ITD upstream of BTK in AML and show that the BTK inhibitor ibrutinib inhibits the survival and proliferation of FLT3-ITD primary AML blasts and AML cell lines. Furthermore ibrutinib inhibits the activation of downstream kinases including MAPK, AKT and STAT5. In addition we show that BTK RNAi inhibits proliferation of FLT3-ITD AML cells. Finally we report that ibrutinib reverses the cyto-protective role of BMSC on FLT3-ITD AML survival. These results argue for the evaluation of ibrutinib in patients with FLT3-ITD mutated AML.

Acute myeloid leukaemia (AML) is primarily a disease of the elderly with a median age at diagnosis of 72 years. Many elderly patients tolerate current intensive cytotoxic chemotherapy regimens poorly and therefore treating the older less fit patient with AML presently remains challenging^1^. Accordingly despite considerable improvements in the outcomes for younger fitter patients with AML over the past 50 years we have seen little improvement in survival for the majority group of older patients with the disease. It is envisaged that improvements in survival for all patients with AML will eventually come from targeted therapies that evolve from an improved understanding of the biology of the disease.

Drug targeting of pro-tumoral tyrosine kinases has resulted in considerable progress in outcomes for patients with chronic myeloid leukaemia^2^, chronic lymphocytic leukaemia and mantle cell lymphoma^3^,^4^. Furthermore tyrosine kinase inhibition in these diseases is associated with a favourable side effect profile, which has permitted successful use in both younger and older patients alike. A number of receptor and non-receptor tyrosine kinases have been identified as functionally important in the biology of acute myeloid leukaemia (AML)^5^,^6^,^7^. Protein Kinase B (AKT), phosphatidylinositol 3-kinase isoform p110delta (P13-K), Signal Transducer and Activator of transcription 5 (STAT5), Mitogen-Activated Protein Kinase (MAPK) and Bruton’s tyrosine Kinase (BTK), have all been shown to be part of pathways that regulate AML survival^8^,^9^,^10^,^11^.

Various receptor tyrosine kinase mutations have been identified in AML patients^10^. 20% of patients with AML are affected by internal tandem duplication (ITD) of the juxtamembrane region of the FMS-like tyrosine kinase-3 receptor (FLT3)^12^,^13^,^14^. The activating FLT3-ITD mutations in AML regulate downstream pro-leukaemic pathways^15^ making FLT3 an attractive drugable target in this disease^16^. However to date drugs targeting FLT3 have demonstrated limited clinical efficacy suggesting that FLT3 inhibitors alone are unlikely to be effective^17^, and that other downstream targets in this pathway may be more relevant.

Bruton’s tyrosine kinase (BTK) is a non-receptor tyrosine kinase which is functionally important in a spectrum of benign and malignant haematopoietic cells of both the lymphoid and myeloid compartments^18^,^19^,^20^,^21^,^22^. Recently the oral BTK inhibitor ibrutinib has been shown to inhibit AML blast proliferation, migration and leukaemic cell adhesion to bone marrow stromal cells in approximately 80% of primary samples tested, leading to the initiation of early phase clinical trials of ibrutinib in AML^9^,^23^. The anti-proliferative effects of BTK inhibition in human AML are mediated via inhibition of downstream AKT, MAPK, ERK and Nuclear Factor-KappaB (NF-κB) pro-survival signalling however the upstream drivers of BTK activation in human AML have yet to be fully characterised.

In this study we place BTK activation downstream of mutated FLT3 in primary AML cells and furthermore show how inhibition of BTK (by ibrutinib and RNA interference) targets FLT3 mutated AML cells by inhibiting cell survival. We also report how ibrutinib synergises in combination with daunorubicin, and how ibrutinib functions in part by reducing the cyto-protection provided to FLT3-ITD AML cells by bone marrow stromal cells. Here we provide a biologic rationale for the targeting of BTK in FLT3 mutated AML.

## Materials and Methods

### Materials

Anti-phosphorylated and total FLT3, AKT, BTK, STAT5 and MAPK antibodies were purchased from Cell Signalling Technology (Cambridge, MA). Anti-CD34-PE, anti-CD90-FITC, anti-CD73-PE, anti-CD105-APC antibodies were purchased from Miltenyi Biotec (Auburn, CA, USA). Ibrutinib was obtained from Selleck Chemicals. All other reagents were obtained from Sigma-Aldrich (St Louis, MO, USA), unless indicated.

## Methods

### Cell lines and primary cells

The AML-derived cell lines were obtained from the European Collection of Cell Cultures and DMSZ where they are authenticated by DNA-fingerprinting. In the laboratory they are used at low passage number for a maximum of 6 months post-resuscitation, testing regularly for Mycoplasma infection. AML blasts were obtained from patients’ bone marrow or blood following informed consent and under approval from the UK National Research Ethics Service (LRECref07/H0310/146). Moreover, all aspects of the study were carried out in accordance with the approved guidelines. For primary cell isolation, heparinized blood was collected from patients and human peripheral blood mononuclear cells (PBMCs) isolated by Histopaque density gradient centrifugation. AML samples that were less than 80% blasts were purified using the CD34 positive selection kit. FLT3-ITD analysis was performed by the haemato-oncology diagnostic service (Cambridge University Hospitals NHS Trust, Cambridge, UK) as previously described and 5 patients with FLT3-ITD and 6 with no FLT3 mutation were selected for this study^14^,^24^.

Human bone marrow stromal cells (BMSC) were isolated from bone marrow aspirates of AML patients. Mononuclear cells were collected by gradient centrifugation and plated in growth medium containing RPMI and 20% FBS and 1% l-glut. The non-adherent cells were removed after 2 days. When 60%–80% confluent, adherent cells were trypsinised and expanded for 3–5 weeks. BMSCs were checked for positive expression of CD105, CD73, and CD90 and the lack of expression of CD45 and CD34 by flow cytometry as previously described^9^.

### Western immunoblotting

Sodium dodecyl sulfate-polyacrylamide gel electrophoresis and Western blot analyses were performed as described previously. Briefly, whole cell lysates were extracted and sodium dodecyl sulfate-polyacrylamide gel electrophoresis separation performed^25^. Protein was transferred to nitrocellulose and Western blot analysis performed with the indicated antisera according to their manufacturer’s guidelines.

### Survival and apoptosis assays

Cells were treated with different doses of ibrutinib then viable numbers measured with CellTiter-Glo (Promega, Southampton, UK). Flow cytometry for measuring apoptosis was performed on the Accuri C6 flow cytometer (BD Biosciences, Oxford, UK). Samples were collected and stained with annexin- V and propidium Iodide (PI) (Abcam, Cambridge, UK), followed by detection. For the AML-BMSC co-cultures AML cell viability was measured using flow cytometry. After exclusion of BMSC by electronic gating using negative for CD45 expression the extent of AML cells apoptosis was measured using annexin-V.

### Transfections

AML cell line transfections were carried out with 1 × 10^6^ cells using the Amaxa Nucleofactor Kit II. Control siRNA and BTK siRNA 1–3 were purchased from Invitrogen were transfected in at a concentration of 50 nm. For all gene expression experiments the cells were incubated for 24 hours post transfection before RNA extraction. For the Western blot and cell viability assays AML cell were incubated for 48 hours post transfection.

### RNA extraction and real time PCR (RT-PCR)

Total RNA was extracted from 1 × 10^6^ cells by use of the Nucleic acid PrepStation from Applied Biosystems, according to the manufacturer’s instructions. Reverse transcription was performed using the RNA PCR core kit (Life Technology, Paisley, UK). Real-time PCR primers for glyceraldehyde-3-phosphate dehydrogenase (GAPDH) and BTK were purchased from Invitrogen. Relative quantitative real-time PCR used SYBR green technology (Roche, Burges Hill, UK) on cDNA generated from the reverse transcription of purified RNA. After pre-amplification (95 °C for 2 minutes), the PCRs were amplified for 45 cycles (95 °C for 15 seconds and 60 °C for 10 seconds and 72 °C for 10 seconds) on a 384-well LightCycler 480 (Roche, Burges Hill, UK). Each mRNA expression was normalized against GAPDH mRNA expression via use of the standard curve method.

### Statistical analyses

Mann-Whitney test was performed to assess statistical significance from treated sample compared to controls. Results with P < 0.05 were considered statistically significant (*). Results represent the median and in some instances mean ± SD of 3 independent experiments. For Western blotting, data are representative images of 3 independent experiments.

## Results

### Ibrutinib inhibits survival of FLT3-ITD positive AML blasts and AML cell lines

**Figure 1:**
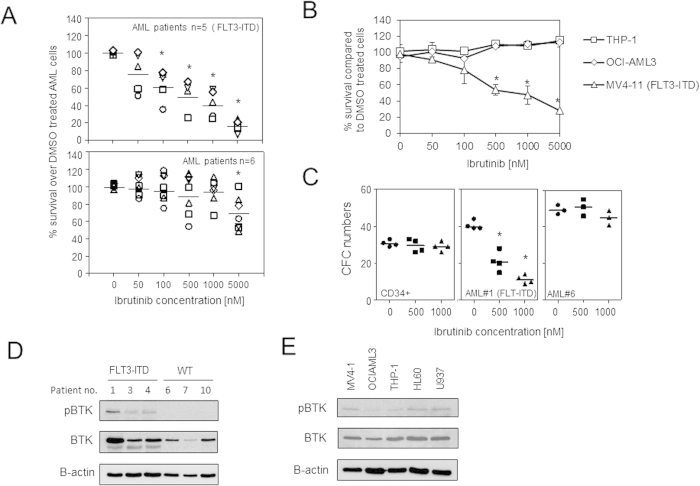
Ibrutinib inhibits survival of FLT3-ITD positive AML. Primary AML blasts from 5 patients with FLT3-ITD and 6 patients with non FLT3-ITD were treated with increasing doses of ibrutinib for 1 hour and then washed and cultured for 72 hours and then assessed by CellTiterGlo. Data were normalised to DMSO treated cells. Line indicates median and *indicates statistical significance of P < 0.05 using the Mann-Whitney test comparing ibrutinib treated samples compared to control. (**B**) AML cell lines which were FLT3-ITD (MV4–11) and non FLT3-ITD (OCI-AML3 and THP-1) were treated with increasing doses of ibrutinib for 1 hour and then washed and cultured for 72 hours and then assessed by CellTiterGlo. *indicates statistical significance of P < 0.05 using the Mann-Whitney test comparing ibrutinib treated samples compared to control. (**C**) AML blasts and CD34+ control cells were treated with 0, 500 and 1000 nM of ibrutinib and colony forming assays were performed to show the number of colonies In all panels line indicates the median and *indicates statistical significance of P < 0.05 between ibrutinib treated groups and control using the Mann-Whitney test. (**D**) Untreated primary AML blasts which were FLT3-ITD and non FLT3-ITD and (E) untreated AML cell lines were examined for the expression of pBTK, total BTK and β-actin. The presented blots were derived from multiple gels. The membranes were cut based on molecular weights and probed with the antibody of interest.

We have previously shown that ibrutinib inhibits factor induced AML blast proliferation and downstream AKT and MAPK signalling pathways^9^. Here we looked to characterise whether FLT3-ITD mutated primary AML and AML cell lines, which accounts for approximately 20% of all AML^14^, responds to BTK inhibition by ibrutinib treatment. First we identified 5 AML patients with FLT3-ITD mutation and 6 AML patients with no FLT3 mutation. Next we treated the primary AML blast with increasing concentrations of ibrutinib for 48 hours followed by analysis of cell survival. AML with FLT3-ITD mutations show increased sensitivity to increasing doses of ibrutinib compared to non-FLT3 mutated AML (Fig. 1A), Similar experiments in AML cell lines shows that MV4–11 (FLT3-ITD), but not OCI-AML3 or THP-1 (non- FLT3-ITD), have reduced survival in response to increasing concentrations of ibrutinib up to a maximum of 5000 nM (Fig. 1B). Finally we used colony forming cell assay to determine the effect to ibrutinib on FLT3-ITD primary AML colony formation compared to normal CD34+ HPC and non-FLT3 mutated AML blasts. Fig. 1C shows that AML with FLT3-ITD showed increased sensitivity to ibrutinib compared to normal CD34+ HPC and non-FLT3 mutated AML. Supplementary figure 1 shows that our positive control AC220 can also inhibit proliferation in MV4–11 and FLT-ITD primary AML. Moreover, AC220 in combination with ibrutinib showed no additive inhibitory effect on MV4–11 suggesting that these inhibitors are working through the same pathway (supplementary figure 2). To confirm the expression of pBTK in human FLT3-ITD AML compared to AML blasts without mutation we examined pBTK and total BTK in primary AML and AML cell lines. Figure 1D shows that pBTK is highly expressed in FLT3-ITD AML compared to no FLT3-ITD mutation. Furthermore, analysis of pBTK in AML cell lines confirms that FLT3-ITD mutated cells have high pBTK expression (Fig. 1E). However we did observe expression of pBTK in non-FLT3 mutated AML cell lines. Together these results suggest that ibrutinib is particularly effective at inhibiting FLT3-ITD mutated AML cell survival.

### Ibrutinib inhibits downstream signalling in FLT3-ITD AML

**Figure 2:**
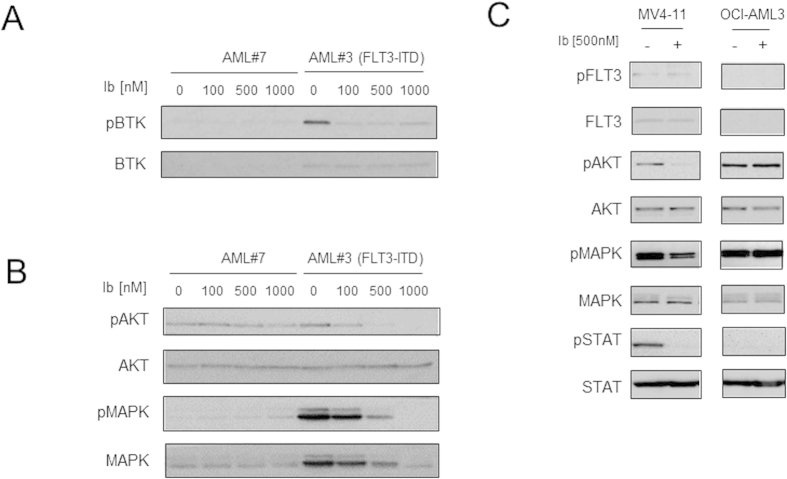
Ibrutinib inhibits downstream FLT3 signalling. (**A**) Primary AML blasts which were FLT3-ITD and non FLT3-ITD were treated with increasing doses of ibrutinib for 3 hour and then whole cell extracts were prepared and Western blot analysis was conducted for pBTK, BTK. and (**B**) pAKT, AKT, pMAPK and MAPK protein levels. (**C**) AML cell lines which were FLT3-ITD (MV4–11) and non FLT3-ITD (OCI-AML3) were treated with 500 nM of ibrutinib for 3 hour and then whole cell extracts were prepared and Western blot analysis was conducted for pFLT3, FLT3, pAKT, AKT, pMAPK, MAPK, pSTAT and STAT protein levels. The presented blots were derived from multiple gels. The membranes were cut based on molecular weights and probed with the antibody of interest.

To determine if ibrutinib inhibits downstream signalling in FLT3-ITD mutated primary AML blasts, we compared AML with FLT3-ITD to AML without FLT3-ITD in response to increasing concentrations of ibrutinib for 3 h. Figure 2A shows that in primary AML associated with FLT3-ITD mutations showed decreased expression of pBTK in response to ibrutinib when compared to total BTK expression. Figure 2B shows that ibrutinib inhibits downstream phosphorylated proteins, including AKT and MAPK in primary AML cells with FLT-ITD. Comparing AML cell lines, MV4–11 (FLT3-ITD) but not OCI-AML3 (non-FLT3-ITD) show reduced activity of downstream phosphorylated AKT, MAPK and STAT5 in response to ibrutinib treatment (Fig. 2C). Moreover, to determine if ibrutinib can target FLT3 directly we examined the effect of ibrutinib on FLT3 phosphorylation and Fig. 2C shows that ibrutinib does not target FLT3 directly. Supplementary Figure 3 shows that AC220 can also inhibit pSTAT5 in MV4–11 cells. Together, these results confirm that ibrutinib inhibits FLT3 mutation associated downstream signalling in FLT3-ITD AML.

### Ibrutinib enhances daunorubicin induced apoptosis

**Figure 3:**
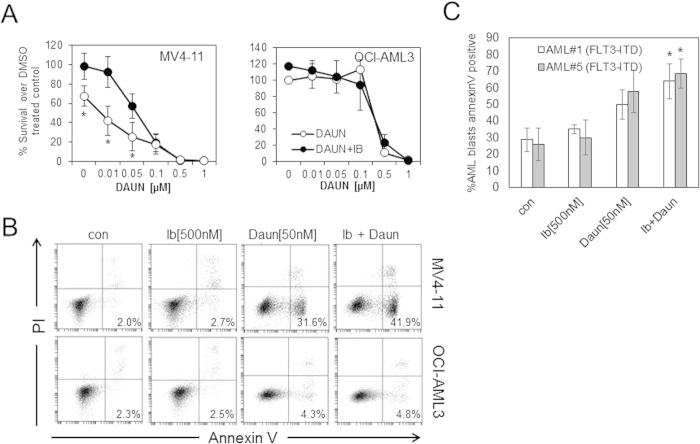
Ibrutinib enhances daunorubicin induced apoptosis. (**A**) FLT3-ITD (MV4–11) and non FLT3-ITD (OCI-AML3) cells were treated with 500 nM of ibrutinib for 1 hour and washed and daunorubicin was added in increasing doses and then cultured for 48 hours and then assessed by CellTiterGlo. (**B**) FLT3-ITD (MV4–11) and non FLT3-ITD (OCI-AML3) cells were treated with 500 nM of ibrutinib for 1 hour and washed and daunorubicin added in increasing doses and then cultured for 24 hours and then assessed by annexin V and PI staining. (**C**) Primary AML (FLT3-ITD) were pre-treated with ibrutinib for 1 h and then treated 50 nM daunorubicin and then cultured for 48 hours on BMSC and then assessed by CD45 and annexin V staining. Cells expressed as percent Annexin V positive. *indicates P < 0.05 Mann Whitney test comparing the ibrutinib plus daunorubicin to daunorubicin alone treated samples.

We next examined the effect of ibrutinib in combination with front line AML chemotherapy daunorubicin by using increasing concentrations of daunorubicin, alone and then in combination with Ibrutinib at 500 nM. Figure 3A and B shows that ibrutinib enhances daunorubicin induced apoptosis in FLT3-ITD AML cell line but not non-FLT3-ITD mutated cell lines. From this observation we hypothesise that in FLT3-ITD AML ibrutinib may permit a dose reduction in daunorubicin treatment while maintaining anti-leukaemic cytotoxicity.

We and others have shown that BMSC provide a level of protection for AML blast cells from chemotherapy treatments^9^,^14^,^23^,^26^ therefore we assessed the efficacy of ibrutinib at overcoming this stroma associated cyto-protection in combination with daunorubicin treatment. Primary AML blasts (FLT3-ITD) when co-cultured on BMSC show increased apoptotic/annexin V positive cells when treated with daunorubicin in combination with ibrutinib (Fig. 3C).

### BTK targeted siRNA inhibits survival of MV4–11 but not OCI-AML3

**Figure 4:**
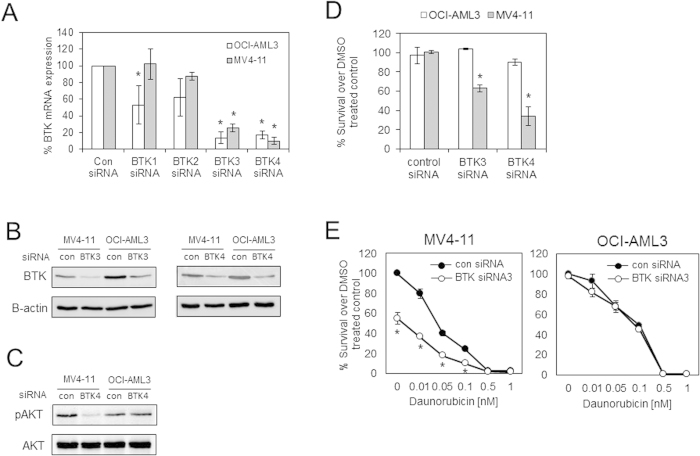
BTK targeted siRNA inhibits survival of MV4–11 but not OCI-AML3. (**A**) FLT3-ITD (MV4–11) and non FLT3-ITD (OCI-AML3) AML cell lines were transfected with control siRNA and 4 BTK siRNA and then cultured for 24 h before RNA extraction analysis for target gene expression using QRT-PCR. (**B**) Western blot analysis for total BTK in response to transfected control, BTK3 and BTK4 siRNA and (**C**) for pAKT and total AKT in response to transfected control and BTK4 siRNA. The presented blots were derived from multiple gels. The membranes were cut based on molecular weights and probed with the antibody of interest. (**D**) FLT3-ITD (MV4–11) and non FLT3-ITD (OCI-AML3) cells were transfected with control siRNA and BTK siRNA3 and 4 and then cultured for 48 h and then assessed by CellTiterGlo. (**E**) FLT3-ITD (MV4–11) and non FLT3-ITD (OCI-AML3) cells were transfected with control siRNA and BTK siRNA3 and then cultured for 24 h and then daunorubicin was added in increasing doses and then cultured for a further 48 hours and then assessed by CellTiterGlo. *Indicates P < 0.05 Mann Whitney test comparing the BTK siRNA transfected samples to control siRNA samples.

It has been reported that ibrutinib has other kinase targets^27^. Therefore to establish an anti-BTK role for ibrutinib in FLT3-ITD AML we evaluated the survival of AML cell lines using BTK KD siRNA experiments. Figure 4A and B show the BTK mRNA and protein expression of AML cell lines, after transfection with siRNA targeted to different parts of the BTK gene. It shows that BTK siRNA3 and 4 are the most effective at BTK gene knockdown in both MV4–11 (FLT3-ITD) cells and OCI-AML3 (non-FLT3-ITD) mutated cells. Figure 4C shows that BTK siRNA4 can inhibit downstream pAKT in MV4–11 but not OCI-AML3. BTK-KD with BTK siRNA3 and 4 compared to control siRNA results in decreased cell survival in MV4–11 (FLT3-ITD) but not in OCI-AML3 (non-FLT3-ITD) mutated cells (Fig. 4D). Finally, in results similar to those observed with ibrutinib, BTK-KD with siRNA3 enhanced daunorubicin induced cell death (Fig. 4E). These data confirm that BTK plays a key role in the survival of MV4–11 FLT3-ITD mutated cells.

## Discussion

The FLT3-ITD mutation is present in circa 20% of patients with AML^12^,^13^,^14^, and furthermore its presence in standard risk cytogenetic AML confers an inferior prognosis in response to treatment with intensive cytotoxic chemotherapy^28^,^29^. FLT3 is a member of the platelet-derived growth factor receptor (PDGF-R) subfamily of receptor tyrosine kinases and is functionally important in haematopoietic progenitor cells^30^. Furthermore the FLT3-ITD represents a driver mutation in human AML^31^. Taken together the FLT3-ITD mutation and its downstream signalling represent a valid therapeutic target.

To date a number of FLT3 inhibitors have been assessed *in vitro* and *in vivo*. The FLT3 inhibitor AC220 (quizartinib) has shown good efficacy in *in vitro* studies^32^. In patients with relapsed AML initial response rates appear high (circa 50%) in keeping with the biologic importance of this pathway. However results of AC220 in relapsed AML have thus far failed to demonstrate significant long-term disease free survival in the absence of consolidative allogeneic transplant. CEP701 (Lestaurhas) has been assessed in clinical studies of relapsed AML but likewise clinical responses when seen appeared short lived and failed to result in long-term disease free survival^33^,^34^,^35^. Similarly PKC412 (Midostaurin) exhibits clinical activity in patients with FLT3 mutation but despite significant numbers of patients with a reducing blast count the drug has no patients which have achieved complete response^36^. The limited clinical success of FLT3 inhibition to date has been attributed to a failure to achieve sufficient FLT3 inhibition in patients. This may be occurring through a number of mechanisms including increased plasma clearance of the drug and the presence of point mutations in the kinase domain or ATP binding pocket of FLT3, which confer drug resistance^17^. More potent FLT3 inhibitors are now in development and under evaluation for the treatment of AML to see if these results can be improved upon. However an alternative strategy would be to target other kinases downstream of the FLT3 mutations.

BTK is a non-receptor tyrosine kinase that belongs to the Tec kinase family and plays an important role in both benign and malignant cells of the haematopoietic system^9^,^37^,^38^,^39^,^40^. BTK is an essential mediator of B-cell receptor (BCR) signalling and is therefore fundamental to the development of normal B-cells^41^,^42^. However other receptors including Toll-like receptors (TLRs) also appear to signal through BTK in some non-lymphoid cells^43^. In the BCR signalling pathway BTK is positioned early on along with three other non-receptor tyrosine kinases PI3-K, LYN and SYK^44^. LYN and SYK have been shown to activate BTK in B cells^45^,^46^. Furthermore LYN and SYK have also been identified as important in AML survival signalling^5^,^47^ and BTK activation via phosphorylation is evident in the blasts from the majority of patients with AML^9^. In addition, it is becoming apparent that ibrutinib inhibits kinases other than BTK including mutant epidermal growth factor receptor and interleukin-2-inducible T-cell kinase^48^,^49^. However, we and others have shown that BTK KD using siRNA or shRNA mimics the ibrutinib effects in AML^50^. Therefore, with the advent of ibrutinib, BTK is a drugable target in AML however the upstream drivers of BTK activation in AML are presently not well characterised.

In this study we demonstrate BTK is activated downstream of the FLT3-ITD in AML and that BTK inhibition targets FLT3-ITD induced survival and proliferation pathways including AKT, STAT5 and MAPK. In addition when we used the FLT3 inhibitor AC220 in combination with ibrutinib there was no additive effect on AML survival, thus supporting the hypothesis that these two inhibitors are working on the same survival pathway/s in FLT3-ITD AML. Furthermore we have shown that treatment of FLT3-ITD AML with ibrutinib or BTK knockdown in combination with daunorubicin increases tumour apoptosis over daunorubicin alone. We show *in vitro* that ibrutinib permits dose reduction of daunorubicin to achieve a given rate of cytotoxicity. As daunorubicin toxicity presents a clinical challenge in the treatment of older less fit patients with AML ibrutinib may allow protocols in the future to dose reduce daunorubicin while maintaining clinical efficacy but improving tolerability. This may result in effective but reduced dose daunorubicin containing treatment regimens being offered with curative intent to patients not currently deemed fit enough for a standard daunorubicin/cytarabine intensive schedule.

Resistance to FLT3 inhibitors can occur through mutations at the drug binding site in the target molecule^31^,^51^. Resistance to ibrutinib has been observed in a small number of patients with CLL through acquired mutations in the BTK drug binding site and gain of function mutations in the down-stream signalling molecule PLCgamma2^52^. If ibrutinib shows clinical efficacy in trials in AML it seems likely that similar resistance mechanisms will be seen in a subset of the patients treated. Clinical studies will ultimately determine the efficacy and duration of response of ibrutinib containing regimens in AML and only thereafter will they establish the frequency of drug resistance and its mechanisms.

In summary this study links a common genetic mutation found in AML to a specific clinically available downstream kinase (BTK) inhibitor, ibrutinib. Furthermore we have identified the potential efficacy of combination therapy with daunorubicin.

## Additional Information

**How to cite this article**: Pillinger, G. *et al*. Targeting BTK for the treatment of FLT3-ITD mutated acute myeloid leukemia. *Sci. Rep*. **5**, 12949; doi: 10.1038/srep12949 (2015).

## Supplementary Material

Supplementary Information
