# Supplementary material for: Targeting BTK for the treatment of FLT3-ITD mutated acute myeloid leukemia
Source: Sci Rep. 2015 Aug 21;5:12949. doi: 10.1038/srep12949 (PMC4544001; doi:10.1038/srep12949)

Targeting BTK for the treatment of FLT3-ITD mutated acute myeloid leukemia

^1^Genevra Pillinger, ^1^Amina Abdul-Aziz, ^1^Lyubov Zaitseva, ^3^Matthew Lawes, ^2^David J MacEwan,^1,3^Kristian M Bowles* and ^1^Stuart A Rushworth*.

^1^Department of Molecular Haematology, Norwich Medical School, University of East Anglia, Norwich Research Park, Norwich, NR4 7TJ, United Kingdom

^2^Department of Molecular and Clinical Pharmacology, Institute of Translational Medicine, University of Liverpool, Liverpool, L69 3GE, United Kingdom.

^3^Department of Haematology, Norfolk and Norwich University Hospitals NHS Trust, Colney Lane, Norwich, NR4 7UY, United Kingdom

Supplementary Figures 1-12


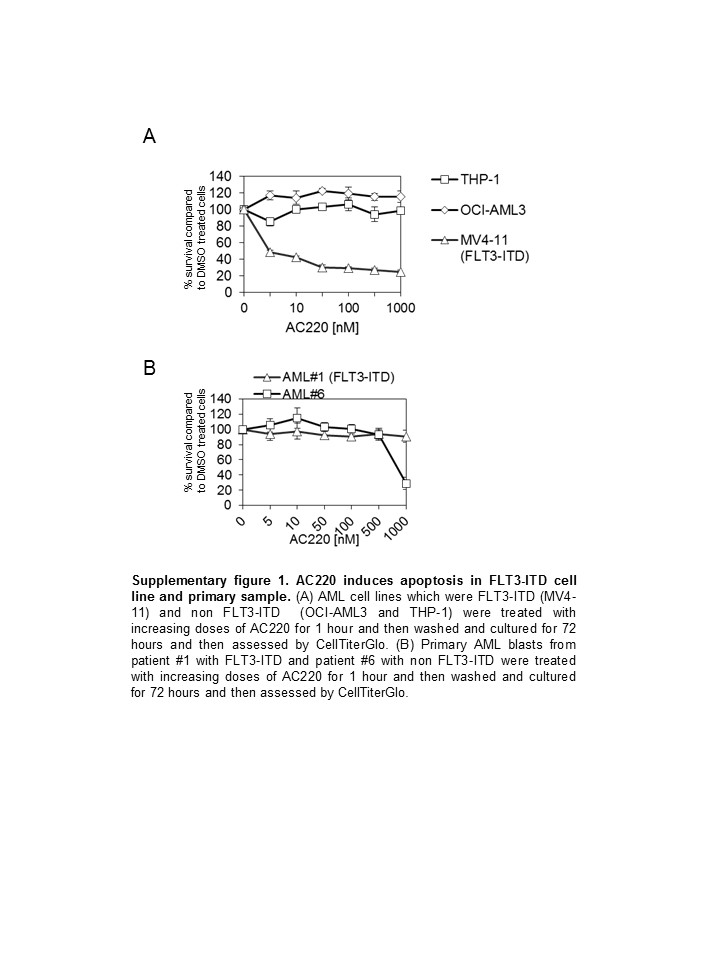

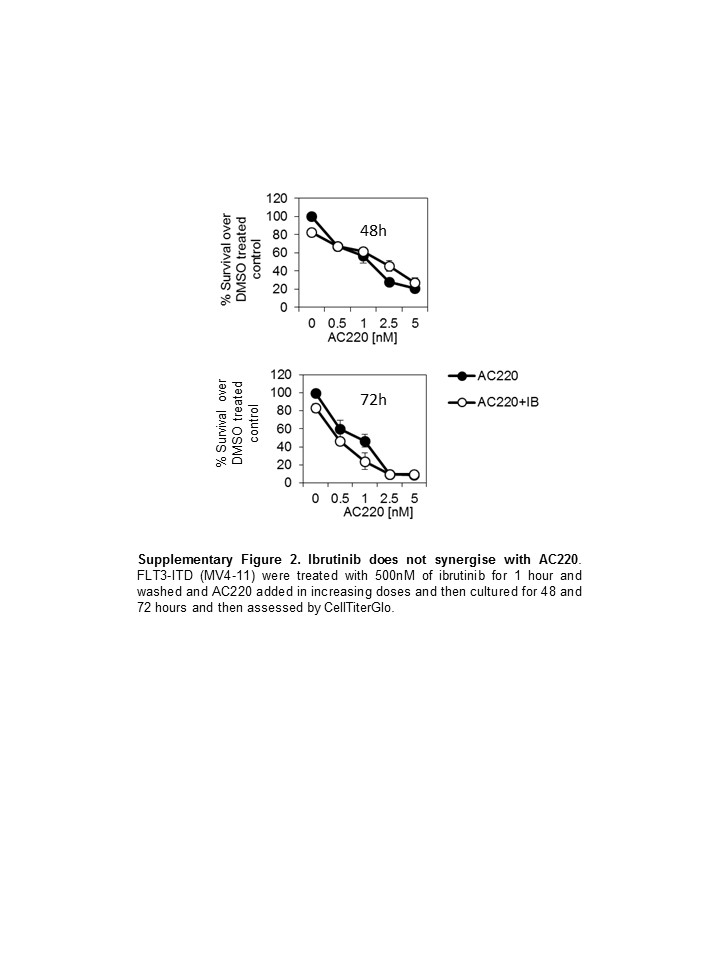

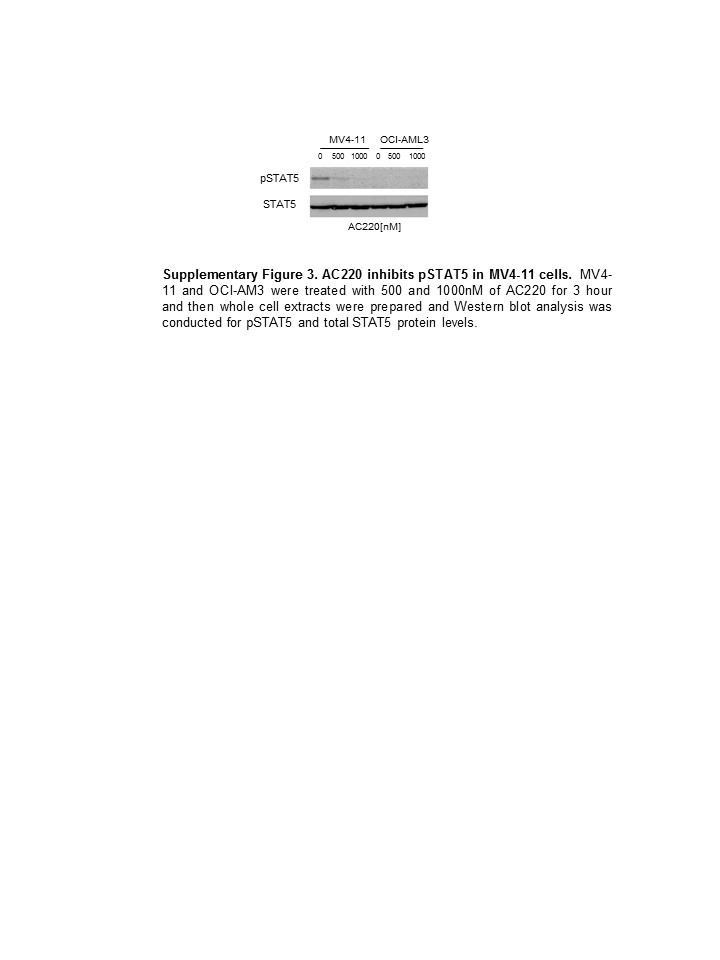

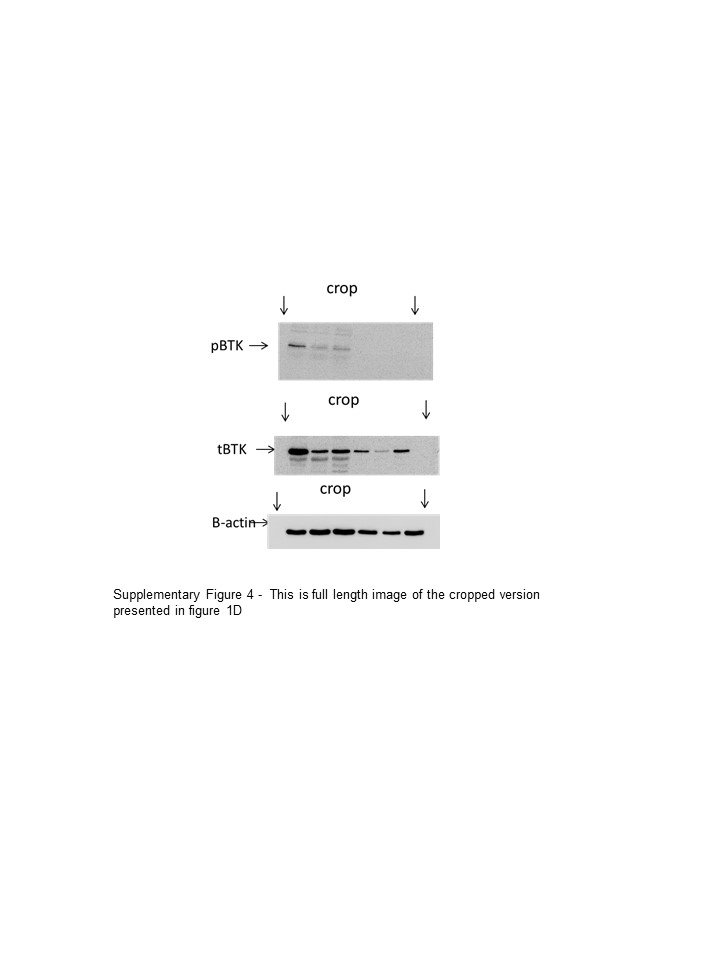

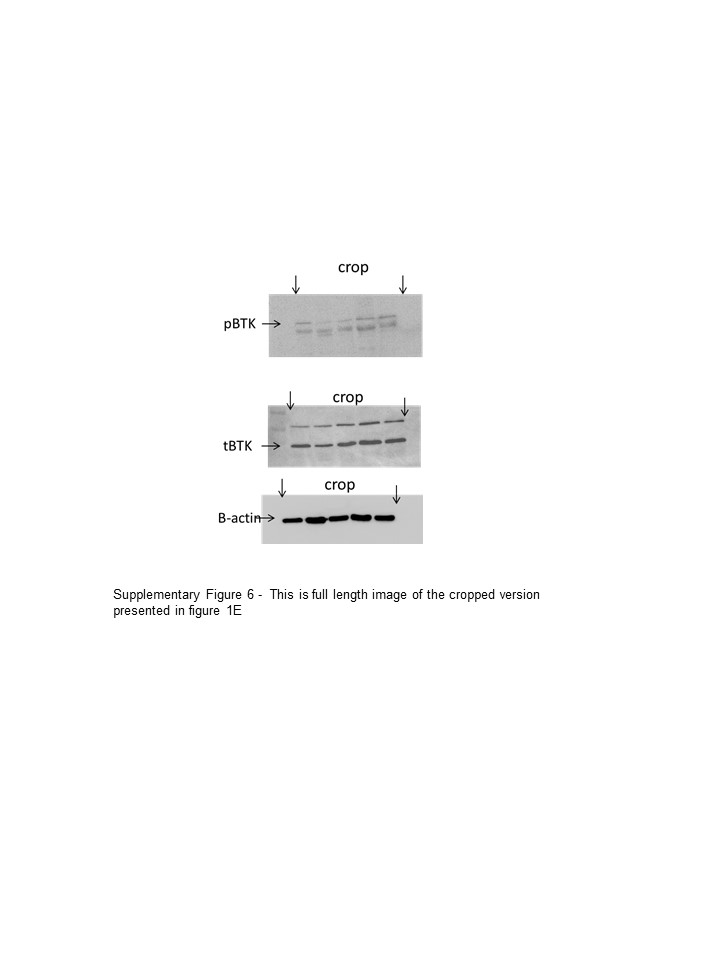

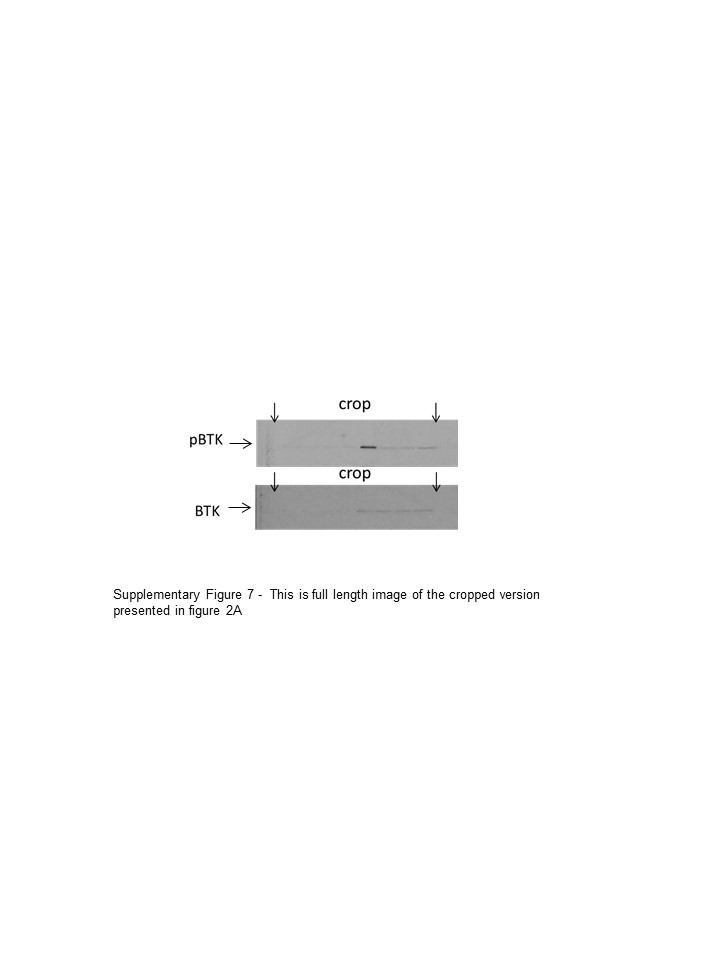

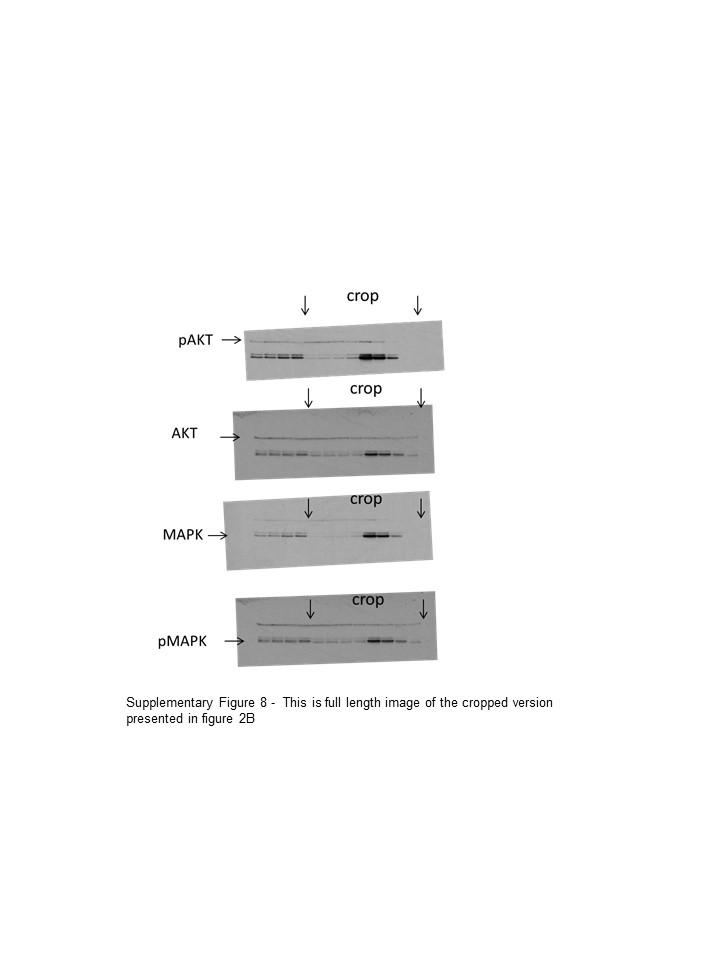

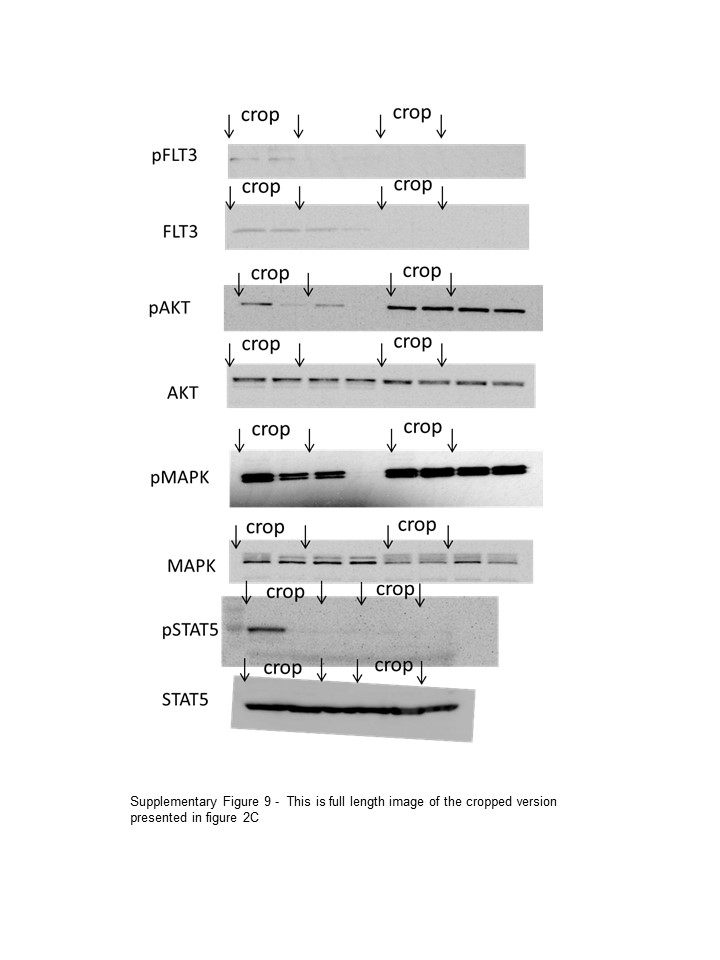

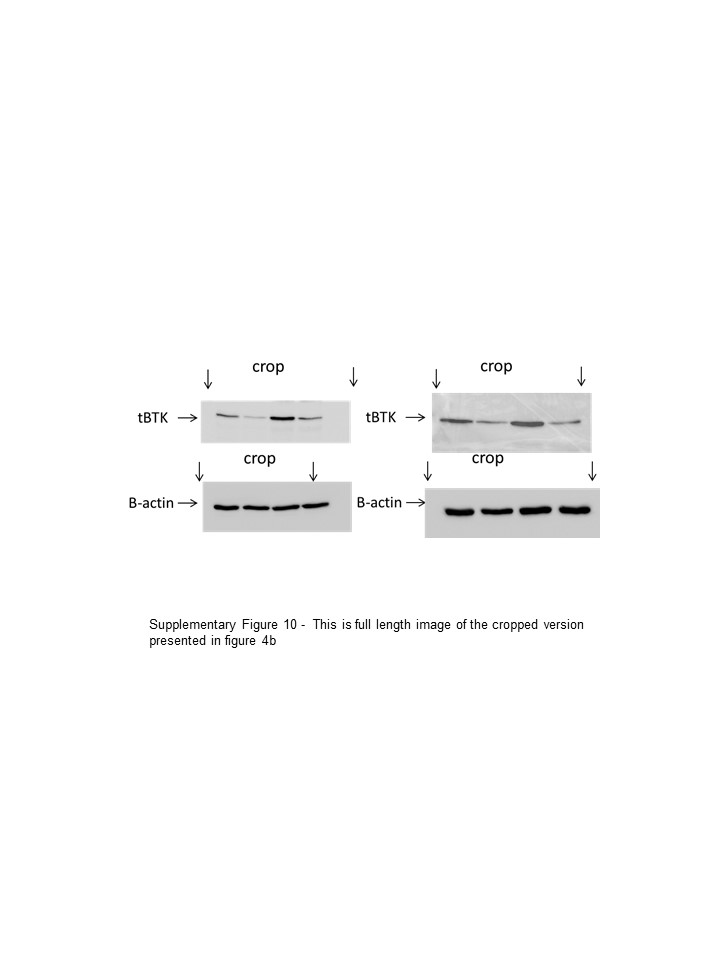

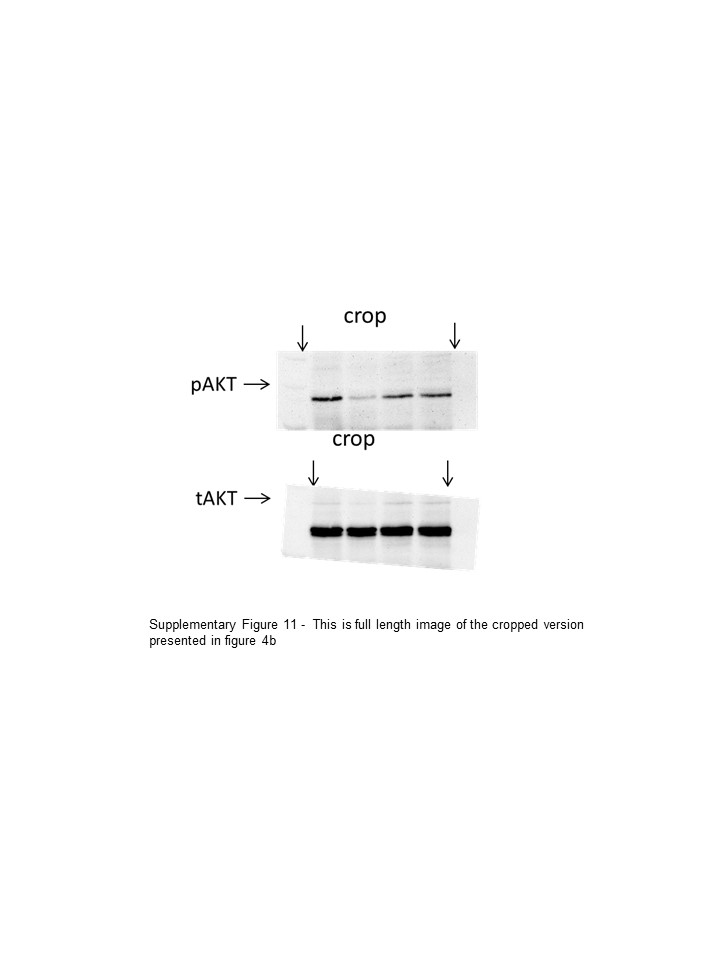

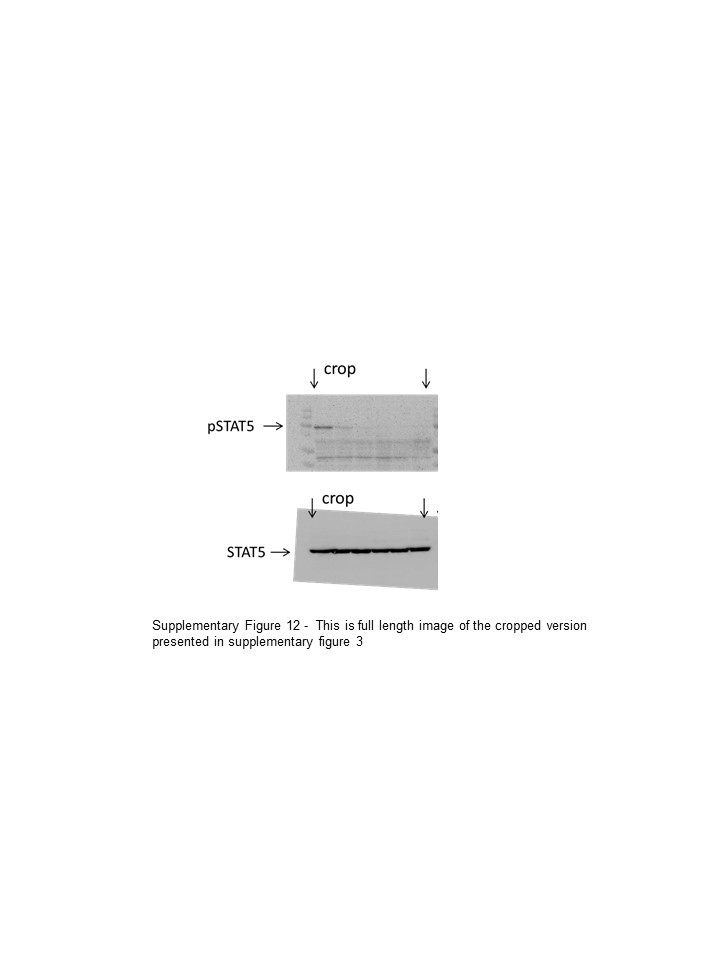

Supplement: Supplementary Information [file srep12949-s1.doc]
